# Assessing the stability of tactical learning in youth footballers: the contribution of a metacognitive double-pass method

**DOI:** 10.3389/fcogn.2026.1664983

**Published:** 2026-03-05

**Authors:** Dimitri Acoumambo, Bachir Zoudji, Hatem Belhouchet

**Affiliations:** 1 Université Polytechnique des Hauts-de-France, Valenciennes, France; 2 Societies and Humanities Research Laboratory, Valenciennes, France; 3 Institute of Transversalities, Sports and Health, Valenciennes, France

**Keywords:** declarative recognition, development, double pass, football, metacognition, tactical stability

## Abstract

The stability of tactical learning in youth football remains insfficiently explored. This study examined players' explicit recognition of taught tactical content and the short-term stability of these judgements using a metacognitive double-pass (DP) method. A total of 225 youth footballers (U-13, U-15, U-18; *n* = 75 per group) completed both a single-pass (SP) and a delayed double-pass DP assessment across 40 standardized animated tactical scenarios. Results revealed a clear decline from SP to DP, suggesting overestimation in immediate (SP) judgements. Although stability improved with age, it did not approach high declarative integration, even in U-18s. These findings support the use of DP method as a conservative indicator of declarative accessibility and underline the importance of instruction that fosters guided verbalisation, reflective practice, and metacognitive monitoring. The double-pass approch thus appears to be a valuable tool for refining formative assessment in sport contexts.

## 1 Introduction

Within youth football training, the stability of tactical learning remains insufficiently studied. Yet the consolidation of tactical knowledge is essential for producing contextually appropriate decisions during match play (Williams and Hodges, 2005; Memmert and Raabe, 2018). Despite extensive work on tactical skill acquisition, little is known about its retention and players' explicit recognition. This gap is particularly noteworthy given that contemporary pedagogical approaches place a strong emphasis on autonomy and metacognitive processes as core components of learning in sport (Flavell, 1979; Veenman et al., 2006; Zimmerman, 2002). These frameworks assume that players can both apply knowledge in action and explicitly recognize and evaluate taught content. The literature distinguishes two complementary forms of tactical knowledge declarative and procedural (Anderson, 1982). Declarative tactical knowledge refers to the explicit understanding of game principles, spatial organization, and collective intentions (McPherson, 1994; Sánchez-López et al., 2022). It includes the ability to identify relevant options and justify their appropriateness with respect to collective principles (Kannekens et al., 2009). In contrast, procedural tactical knowledge reflects the application of this information in action through the selection and execution of behaviors adapted to evolving game conditions (Costa et al., 2011; Palucci Vieira et al., 2019). As described by (McPherson 1994), it relies on integrated decision-making schemas that link cognitive choice with motor execution in real time. However, knowing what to do and being able to deploy it accurately in action do not guarantee that players can reliably estimate the robustness of their own knowledge, metacognitive issue (Koriat, 1997). Recent advances in cognitive science emphasize that the effectiveness of these two forms of knowledge depends partly on metacognitive monitoring, that is, the ability to evaluate the accuracy of one's judgements (Fleming and Dolan, 2012). Closely related is judgement calibration, i.e., the alignment between perceived knowledge and actual performance, a mechanism influenced by task familiarity and developmental maturity (Koriat, 1997). Neuroscientific work suggests that metacognitive monitoring engages prefrontal networks associated with performance evaluation and error awareness, supporting the regulation of confidence under pressure (Fleming and Dolan, 2012; Shea et al., 2014). In parallel, cognitive load theory indicates that tactical complexity can exceed working-memory capacity, particularly in young athletes, thereby constraining the consolidation of declarative anchors (Sweller, 2011). Under competitive conditions, attentional regulation becomes critical to filter relevant cues and prevent cognitive overload (Van Merriënboer and Sweller, 2010). Altogether, the interplay between declarative knowledge, procedural execution, and metacognitive control emerges as a central determinant to tactical performance. Yet these components do not always mature in synchronously, creating transient developmental gaps.

This raises two questions: (1) can young players explicitly recognize taught tactical situations, and (2) is this recognition stable over short intervals within a single session? Although metacognition is key to learning, explicit identification of taught tactical content remains underexplored in sport. This gap limits inferences about depth of learning and memory consolidation, both of which are crucial for the long-term retention and transfer of knowledge during match play (Schmidt and Lee, 2011; Schmidt and Wrisberg, 2008). Because such judgements are context-sensitive, their short-term stability warrants closer examination (Beilock and Carr, 2001). This study is therefore grounded in two key observations: (1) players do not consistently articulate taught tactical content; (2) declarative judgements made in real time, immediately after an action or sequence, may lack stability and be influenced by contextual factors such as engagement level or physical condition, without necessarily reflecting cognitive anchoring. In light of these observations, the present study investigates two fundamental dimensions of tactical learning: (1) the explicit recognition of a situation previously encountered in training; and (2) the short-term stability of this recognition as a conservative proxy for metacognitive judgement strength. Together, these dimensions provide a cross-sectional view of how youth players access tactical memory and mobilize knowledge in a semi-natural setting. Understanding these processes is essential for refining pedagogical practices, adapting instructional methods to players' developmental stages, and designing assessment tools that more accurately reflect genuine learning outcomes. To investigate these dimensions, the study draws on a multidisciplinary theoretical framework that integrates cognitive psychology, sport pedagogy, and developmental science. Our framework integrates (1) work on reflective judgement in sport pedagogy and (2) research on metacognitive stability using within-session double-testing. This conceptual framework underpins the study's methodological design and central hypothesis: explicit recognition and the stability of related judgements increase progressively with age. The following section outlines these theoretical foundations, detailing the proposed mechanisms underpinning the long-term acquisition of tactical knowledge and the stability of its declarative assessment.

### 1.1 Recognition and reflective assessment of tactical learning

Tactical learning in young footballers is typically studied from two dominant perspectives, the cognitive experimental and the ecological, which often overlook the explicit recognition and self-evaluation of taught content (Abernethy et al., 2005; Gumusdag et al., 2025; Zoudji et al., 2010). (2) The ecological approach, which emphasizes adaptive regulation within the player-environment interaction (Araújo et al., 2006; Chow and Kee, 2022). Yet both paradigms frequently neglect a crucial dimension: the player's ability to explicitly recognize and assess the tactical content introduced during training, and to relate it to their own performance. This reflective capacity constitutes a key indicator of meaningful internalization.

Recent studies, in sport pedagogy, suggest that pedagogical that Tactical Games for Understanding (TGfU) approach encourage metacognitive engagement in young athletes (Light, 2004; Harvey and Jarrett, 2014). These behaviors can take the form of verbalisation, explicit recognition of training-related action, or introspective judgements about one's own decisions during play. They reflect a form of reflective judgement, in which players evaluate their choices in light of the tactical principles discussed during prior instruction. Explicit recognition and brief self-evaluation mark a shift from mere execution to the conscious appropriation of tactical principles (Light, 2004; Harvey and Jarrett, 2014). This process reflects a deeper level of integration, as it requires the activation of structured declarative memory. Findings from developmental psychology support this trajectory. Among the youngest age group (U-13), verbalisation tends to be fragmented, intuitive, and unstable, whereas in adolescence (U-15 and U-18), players progressively develop the ability to identify, label, and reapply tactical concepts in gameplay, demonstrating of increasingly structured metacognition abilities, whether implicit or explicit. Recognition and reflective judgement may support the consolidation and transfer of tactical principles, highlights the limitations of behavioral observation alone as an indicator of understanding. This is the rationale underpinning our approach, which introduces an evaluation method centered on recognition and declarative metacognitive judgement. The temporal stability of these judgements will be examined in the next section.

### 1.2 Temporal stability of tactical metacognitive judgements

If recognition and reflective judgement serve as indicators of the conscious appropriation of knowledge, they must demonstrate temporal stability to reflect cognitive anchoring. In other words, a player who identifies a tactical action as previously taught should be able to sustain that judgement under comparable, repeated conditions, regardless of fatigue, random variation, or contextual influences. The intra-individual stability of metacognitive judgements thus constitutes a criterion for assessing the durability of learning. This concept, grounded in research from cognitive psychology, rests on the assumption that consistent short-term self-assessments are positively associated with stronger long-term memory representations (Flavell, 1979; Veenman, 2017; Dunlosky and Metcalfe, 2009). Stable judgements across comparable contexts suggest that the learner is reliably accessing declarative knowledge. However, within the domain of sport, this concept remains largely underutilized. Most assessment protocols still rely on immediate performance outcomes or observational data, without accounting for the reliability or short-term stability of self-reported judgements. Recent studies in educational science and sport didactics have shown that immediate metacognitive judgements so-called ‘hot' judgements are often subject to overestimation. They are often influenced by perceived success, familiarity with the context, or the subjective ease of processing (Flavell, 1979; Frank and Kuhlmann, 2017). These judgements are shaped by both immediate internal experiences and pre-existing beliefs about learning, which can result in a significant discrepancy between subjective perception and actual knowledge. This tendency is particularly pronounced in younger children, whose metacognitive development is still in progress. To address this, we propose the use of an intra-session double-testing method. Accordingly, double-pass (DP) does not measure an additional recognition attempt, but the stability of the judgement across repeated exposure. This involves presenting the same tactical situation twice, at short intervals, without informing the players in advance. This implicit protocol enables the measurement of declarative stability in recognition: a player who consistently identifies the same action as having been taught is likely drawing on a stronger and more organized memory than one whose judgement varies. Accordingly, the DP condition should be interpreted as an index of intra-session stability rather than a second recognition performance. This approach aligns with developmental models (Veenman et al., 2006), which suggest that self-regulation, verbalisation, and evaluative accuracy improve with age. Based on this framework, we expect a marked increase in metacognitive stability between the U-13, U-15, and U-18 age groups. These categories mark key developmental stages within academy pathways, where selection, role differentiation, and preparation for senior competition typically occur. Maturation-related factors may, in turn, influence both the recognition of tactical content and the short-term stability of metacognitive judgements (Deprez et al., 2015; Höner and Feichtinger, 2016; Reilly et al., 2000). By combining reflective recognition with its temporal stability, this study aims to deepen our understanding of how tactical knowledge is internalized during training. The DP method provides a novel way of assessing not only what players recognize as having been taught, but also the extent to which their judgements are reliable, structured, and transferable to game contexts.

In light of the theoretical contributions discussed, it is essential to employ a protocol capable of capturing both explicit recognition of taught tactical content and the temporal stability of that recognition. The literature suggests that reflective judgement and stability are complementary dimensions in assessing the depth of learning particularly in intensive training environments, where cognitive load is high and verbalisation is seldom used. To address these demands, the present study adopts an experimental design combining two assessment procedures: a single-pass test (SP) measuring immediate recognition of tactical actions, and a DP test evaluating the intra-session stability of recognition judgements.

This original protocol thus moves beyond single-point assessments by examining the internal consistency of metacognitive judgements over a short time interval. By applying this approach across three distinct age categories (U-13, U-15, U-18), the study aims to assess the influence of cognitive development on the declarative consolidation of tactical learning. The methodology rests on two core hypotheses: (1) older players will demonstrate a greater ability to explicitly recognize previously taught actions; and (2) such recognition will be more stable over time, reflecting stronger memory anchoring and more advanced metacognitive regulation. The following section details the methodological choices made to test these hypotheses, including sample selection, testing procedures, and the development of assessment materials.

## 2 Method

The experimental protocol was designed to assess the depth of tactical learning in young footballers by examining two interrelated dimensions: (1) immediate declarative recognition of taught tactical situations; and (2) intra-session stability of that recognition (consistency of metacognitive judgements). Two experimental conditions were used: (1) a single-pass (SP) condition in which each tactical situation was presented once; and (2) a double-pass (DP) condition in which each situation was reintroduced after a pseudo-random delay of 7-15 intervening trials. Both conditions were applied across three age categories (U-13, U-15, U-18) to examine the influence of age-related development on recognition and stability.

### 2.1 Construction and taxonomy of tactical actions

**Figure 1:**
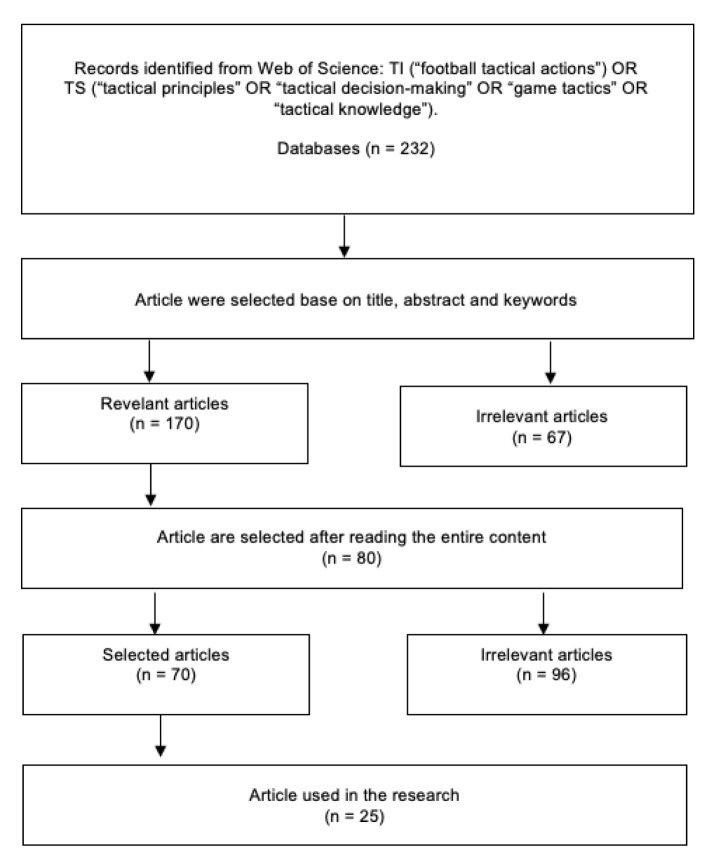
PRISMA flow diagram illustrating the identification and selection of studies on tactical knowledge in youth football.

To operationalise these dimensions, we developed a structured taxonomy of explicitly teachable tactical actions aligned with the ten core FUT-SAT principles and supported by a systematic literature review. The methodological procedure underpinning its construction and validation is presented below. A systematic review was conducted in accordance with PRISMA guidelines (Moher et al., 2009), covering publications from December 2000 to January 2024. The literature search was conducted in the Web of Science database using a combination of keywords, topic fields, and Boolean operators. The search strategy included the following terms: TI (“football tactical actions”) OR TS (“tactical principles” OR “tactical decision-making” OR “game tactics” OR “tactical knowledge”). Inclusion criteria were: (a) be published in peer-reviewed journals; (b) explicitly reference at least one FUT-SAT principle; (c) concern players in the U-13 to U-18 age categories; and (d) be available in English, French, or Spanish. Studies were excluded if they focused solely on performance outcomes without a tactical rationale, lacked methodological or conceptual rigor, or were published before 2000 (except seminal contributions). Of 237 records initially screened, 25 met all inclusion criteria (Figure 1). Tactical actions were retained if they met three criteria: empirical or theoretical support; a decision-making component; and a contribution to collective organization. Each action had to be clearly formulated, appear in at least one included study, and be defined as a deliberate spatiotemporal adaptation involving intentional decisions on movement, positioning, or interaction in response to dynamic constraints (González-Víllora et al., 2015). Actions were classified according to the four tactical levels proposed by (Costa et al. 2011): general, fundamental, operational, and specific. Each action also required observable coordination with at least one teammate toward a collective objective (O'Connor et al., 2018). Frequency of occurrence and execution complexity were excluded as selection parameters due to high variability across players and contexts (Mackenzie and Cushion, 2013). Likewise, motor, cognitive, and contextual complexity could not be systematically evaluated within a purely declarative framework. An initial set of 47 tactical actions was identified and mapped to corresponding FUT-SAT principles. Two UEFA A-licensed or UEFA Pro-licensed coaches specializing in youth development independently evaluated each action using descriptor sheets comprising the action name, a concise definition, the associated principle, and evaluation criteria. They assessed definitional clarity, alignment with the corresponding principle, and compliance with inclusion criteria, and could propose rewording, substitution, or exclusion. Feedback was collected anonymously via an online form and re-evaluated in a second validation round using a Delphi-style consensus procedure (Hsu and Sandford, 2007; Quartiroli, 2025). Following this process, 40 actions were retained; four were excluded for insufficient specificity and three for redundancy. Inter-rater agreement reached 85% (Cohen's κ = 0.78), indicating substantial agreement. The final list appears in Table 1.

**Table 1:** Mapping of the 40 validated tactical actions to the ten principles of the FUT-SAT model (Costa et al., 2011).

**Game phase**	**Tactical principle**	**Tactical action**	**Definition**	**Autors/s**
**Offensive**	**Penetration**: Movement of the player with the ball toward the goal.	Get past	Movement along the touchline to reach a ball from the wing toward a finishing area.	• (Acoumambo and Zoudji 2023)
		Overlapping	Movement between two players, where one passes behind the ball carrier to receive a forward pass.	• (Izquierdo et al. 2023); (Forcher et al. 2023)
	**Offensive cover**: Offensive support for the player with the ball	Support play	Support from a player in front of the ball carrier, to offer a passing solution.	• (Matsubara et al. 2022)
		Support	Support from a player behind the ball carrier, to offer a passing solution.	• (Matsubara et al. 2022)
		Drop off	Movement in which a player moves away from their direct marker or the forward line to create space, receive the ball, or facilitate link-up play	• (Gregory et al. 2022)
	**Depth**: Movement of players between the last defender and the attacker.	Fast attack	Rapid attacking movement forward, with few passes to reach the goal as quickly as possible.	• (Aranda-Malavés et al. 2024)
		Play in the gap	A passing movement in the space between two defenders, to find a free team-mate between the lines.	• (Håland et al. 2020); (Chawla et al. 2017)
		Direct play	An attacking moves forward, into the deep, to take advantage of a defensive imbalance.	• (Plakias et al. 2024)
		Verticality	Rapid attacking movement toward goal, playing along the length of the pitch, with forward passes and deep runs.	• (Chawla et al. 2017); (Cho et al. 2022)
		Open the gap	Move toward an open area to find space to attack.	• (Chawla et al. 2017); (Cho et al. 2022)
		Throw the ball upfield	Action aimed at advancing play toward the opponent's half, often through a long pass, throw, or clearance, to relieve pressure or initiate attack.	• (Han et al. 2024); (Plakias et al. 2024)
	**Width and Length**: Movement of players to expand and use the effective playing space.	Switching play	Action of transferring the ball from one side of the pitch to the other, aiming to exploit space, unbalance the opponent's defensive structure, or accelerate the attacking transition	• (Cho et al. 2022)
		Horizontality	Lateral movement across the width of the pitch, with little forward movement toward the goal.	• (Chawla et al. 2017)
		Decoy run	Movement without the ball in two stages: a call in one direction, then a quick change to stand out in the other direction.	• (Beernaerts et al. 2020)
		Flick-on	A quick, light redirection of the ball, usually with the head or foot, to guide it into a teammate's path without full control	• (Miyamoto and Kaneki 2019)
		Leave one's zone	Movement consisting of leaving the allocated area to adapt to the game situation.	• (Miyamoto and Kaneki 2019)
		Breaking free from a marker	Movement to free oneself from an opponent's markings.	• (Díaz Losquiño and Solà Santesmases 2018)
		Crossing runs	Coordinated attacking movement between two players without the ball, aimed at crossing their trajectories in order to disorganise the defense.	• (Beernaerts et al. 2020)
		Permutation	A move in which two players change positions while retaining the same role in the team's organization.	• (Fernandes et al. 2021)
	**Offensive unit**: Movement of the last line of defenders toward the attacking midfield, to support the offensive actions of team-mates.	Transition	A move in which the team changes its phase of play, moving from attack to defense or defense to attack.	• (Wang et al. 2022)
		Possession of the ball	Movement to keep the ball	• (Ade et al. 2016)
		Placed attacks	Well-constructed attacking action, with several passes and players involved.	• (Leontijević et al. 2021)
		Combination	Action between several players.	• (Muñoz et al. 2024)
		Counter attack	Quick action after a recovery, to take advantage of an unorganized defense.	• (Forcher et al. 2023)
**Defensive**	**Delay**: Actions designed to slow down an opponent's attempt to advance with the ball.	Jockeying the player	Action where a defender delays or controls an opponent's advance by positioning their body to contain movement, without immediately tackling.	• (Moreira et al. 2020)
		Retreating defense	A collective defensive movement in which the team drops back toward its own goal to reduce available space in depth, reorganize its structure, and protect the central defensive zone.	• (Fernandez-Navarro et al. 2016)
		Pressing	Immediate marking action to recover the ball.	• (Miyamoto and Kaneki 2019)
		Defensive retreat	An individual or collective backward movement by defenders aimed at regaining balance and positional security after being displaced or bypassed.	• (Fernandez-Navarro et al. 2016)
		Counter-pressing	The act of pressing your opponent very aggressively as soon as you lose possession of the ball.	• (Miyamoto and Kaneki 2019)
		Shifting defense	A coordinated lateral or diagonal movement of the defensive line or block in response to the ball's movement, aiming to maintain compactness, coverage, and balance.	• (Miyamoto and Kaneki 2019); (Fernandez-Navarro et al. 2016)
	**Defensive cover**: Positioning of off-ball defenders behind the ‘trailing' player, providing defensive support.	Cover the defense	A player positioned lower than his partners to intervene when the ball is lost.	• (Ade et al. 2016); (Fernandez-Navarro et al. 2016)
	**Balance**: Positioning of defenders off the ball in reaction to attacker movements, trying to achieve numerical stability or superiority in the opposition.	Occupy the area	Positioning consisting of occupying an area of the pitch in an organized manner to prevent the opponent from taking any action.	• (Nunes et al. 2022)
		Position	Positioning players on the pitch	• (Miyamoto and Kaneki 2019)
		Positional staggering	Position players at different heights on the pitch to encourage ball movement and maintain team balance.	• (Teixeira et al. 2022); (Beernaerts et al. 2020)
	**Concentration**: Positioning defenders outside the ball to occupy vital space and protect the goal area.	Team block	Positioning a compact team in width and depth to limit the space and intervals available to opponents.	• (Miyamoto and Kaneki 2019)
		Flat back four	Positioning players on the same line, usually to put opposing attackers in an offside position.	• (Konefał et al. 2019)
		Collective shifting	A global team movement across the pitch to maintain horizontal and vertical compactness, ensuring coordinated spacing between units.	• (Miyamoto and Kaneki 2019)
	**Defensive unit**: Positioning of defenders outside the ball to reduce the effective playing space of opponents.	Mixed defense	Defensive positioning combining two types of marking to better control opponents' movements.	• (Miyamoto and Kaneki 2019)
		Man-to-man marking	Positioning that consists of following an opponent to reduce his playing options.	• (Miyamoto and Kaneki 2019)
		Zonal defense	Positioning a defender in a playing area, without focusing on a particular opponent.	• (Miyamoto and Kaneki 2019)

## 3 Participants

A total of 225 youth footballers enrolled in accredited training academies participated in this study. Participants were allocated in equal numbers to three age categories: U-13 (*n* = 75), U-15 (*n* = 75), and U-18 (*n* = 75). Inclusion criteria were as follows (Table 2): (1) ≥2 consecutive years in the academy programme; (2) no training interruption >1 month during the current season; and (3) regular coaching by a UEFA A-licensed or UEFA Pro-licensed coach. Sample size was determined a priori via G^*^Power (v3.1.9.2). A priori power analysis for a mixed 2 × 3 ANOVA (within-subject factor: test type; between-subject factor: age group) indicated adequate sensitivity to detect small-to-medium effects (f = 0.25, α = 0.05, 1–β = 0.80). Ethical approval was obtained from the Institutional Ethics Committee of our university on 12 April 2023 (non-invasive observational protocol; no numerical code assigned). Written informed consent was obtained from all players and their legal guardians, and additional authorization was granted by the coaching staff. All participants were informed of their right to withdraw at any time without justification. The study adhered to the principles of the Declaration of Helsinki.

**Table 2:** Training and experience characteristics by age category.

**Characteristics**	**U-13**	**U-15**	**U-18**
**Number of players**	75	75	75
**Mean age of players (years)**	12.6 ± 4	14.3 ± 6	17.1 ± 4
**Number of years of football practice**	4.6 ± 1	6.3 ± 5	9.1 ± 1.2
**Practical training (weekly hours)**	8 ± 2	10 ± 2	12 ± 2
**Theoretical training (weekly hours)**	2 ± 1.3	3 ± 1.3	3 ± 1.3
**Number of official league and cup matches/years**	20 ± 4	25 ± 4	30 ± 4

Values are reported as arithmetic means ± standard deviations.

### 3.1 Material

**Figure 2:**
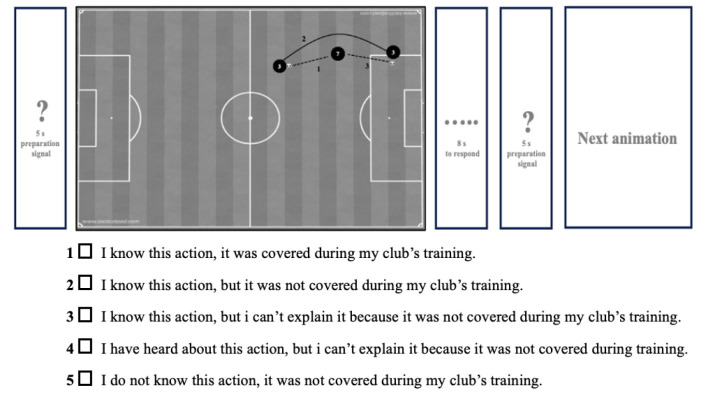
Example of a tactical action animation presented twice in identical form. Solid lines (__________) indicate passes between players (ball movement), while dashed lines (———-) represent player runs (off-the-ball movement). Repetition occurs without visual variation to assess judgement stability rather than perceptual familiarity. Five response options were provided to assess explicit recognition of the tactical action, ranging from full familiarity (1) to complete unfamiliarity (5).

**Figure 3:**
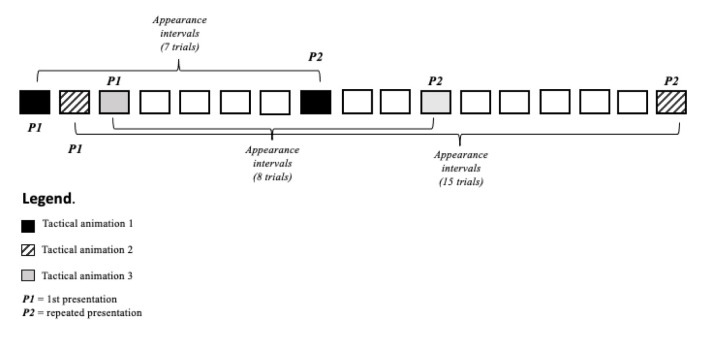
Illustration of the repetition intervals used in DP condition. Each circle represents an animated tactical action. Repeated items were re-introduced after a pseudo-random delay of 7 to 15 intervening items. The pseudo-random spacing reduces temporal predictability, limiting anticipation and practice-related bias in repeated presentations.

The assessment material consisted of 40 tactical actions representative of commonly trained situations, based on the taxonomy developed by (Acoumambo and Zoudji 2023). Each action was rendered as a 2D animation using TacticalPad software (45° oblique angle, 12 frames per second) and validated by two UEFA Pro License coaches. Each animated sequence was followed by five response statements, one of which was a declarative recognition item: “I know this action; it was covered during my training” (Figure 2). In the SP condition, each action was presented only once. In the SP condition, each action was presented only once. In the DP condition, each action appeared twice after a pseudo-random delay of 7 to 15 intervening items (cf. Zoudji and Thon, 2003) (Figure 3).

### 3.2 Procedures and tasks

Sessions were held in an auditorium between May and June 2024, with groups of between 16-22 players. Each session comprised three phases: (1) a brief demographic questionnaire (age, years of practice; Table 1); (2) three practice trials to familiarize players with the response format; and (3) the main sequence of tactical actions. The experimental timeline followed a fixed structure: each animation lasted approximately 6- s, followed by an 8-s response window. An 8-s response window, calibrated in pilot tests with U-13-U-18, balanced reading/decision time with temporal continuity and was applied identically in SP and DP. Inter-item intervals: 1-2 s. Repeated items in the DP condition were reintroduced after 7-15 intervening trials (pseudo-random). All repeated stimuli were identical, ensuring so DP scores index judgement stability rather than additional perceptual learning. Each animation was projected onto a 3 × 4 m screen. At the end of each video, the final frame was held on screen, and players had eight seconds to respond using an interactive keypad system (QuizzBox). Responses could be modified within this time window. The order of presentation of all stimuli was fully randomized, and players were not informed of any repetitions. The lack of motor engagement and brief stimuli limited practice and fatigue effects. No order effects were observed, further supporting the internal validity of the protocol. All responses were automatically recorded in an Excel file. Content validity was supported by expert review (Cohen's κ = 0.78); procedural validity was reinforced by the DP index of intra-session judgement stability.

### 3.3 Data Analysis

#### 3.3.1 Analysis strategy

The statistical analysis was structured to address the two central objectives of the study, as follows. Objective 1: To assess participants' declarative recognition of tactical situations based on age group and test type (SP vs DP). Objective 2: To evaluate the intra-individual stability of metacognitive judgements as an indicator of short-term memory consolidation. All data were processed using IBM SPSS Statistics v27.0. The threshold for statistical significance was set at α = 0.05 for all analyses. Effect sizes were reported using partial eta squared (${\eta^{2}}_{\text{P}}$) for ANOVAs and Cohen's *d* for pairwise comparisons.

**Coding of responses**: For each action, only response option 1 (“I know this action; it was covered during my club's training”) was considered as evidence of explicit recognition. In the SP condition, responses were scored binarily for each action (1 = recognized; 0 = not recognized). In the DP condition, a stability index was calculated for each action. A score of 1 was assigned if the same response (i.e., recognition or non-recognition) was given twice, indicating stable recognition; a score of 0 was assigned if responses were inconsistent. In the DP condition, the score reflects judgement stability rather than a second recognition rate, as only consistent responses across the two presentations are coded as “stable.” Thus, DP serves as an intra-session consistency index rather than a repeated recognition performance.

#### 3.3.2 Statistical analysis

*Main analysis*: interaction between method and age group. A 2 × 3 repeated measures ANOVA was conducted, with test type (SP vs DP) as the within-subjects factor and age group (U-13, U-15, U-18) as the between-subjects factor. Main and interaction effects were examined. Where significant, post hoc comparisons were conducted using Bonferroni-adjusted *p*-values. Main effects and interaction effects were examined. Where significant, post hoc comparisons were performed using Bonferroni-adjusted *p*-values.

Specific intra- and inter-group comparison: Mean comparisons (Student's *t*-tests or univariate ANOVAs) were conducted as follows: (1) Intra-group: within each age category, SP and DP scores were compared to assess the effect of repetition on recognition performance. (2) Inter-group: SP and DP scores were compared across age groups (U-13 vs U-15, U-15 vs U-18, and U-13 vs U-18). Effect sizes were interpreted according to Cohen's (1988) conventions: small (|d| ≈ 0.20), medium (|d| ≈ 0.50), and large (|d| ≈ 0.80). *Dichotomous analysis of declarative integration thresholds:* To refine data interpretation, a dichotomous classification was applied to the scores using two recognition thresholds: (1) Moderate threshold (≥ 0.50): indicating partial or emerging exposure to the tactical action. (2) High threshold (≥ 0.80): indicating frequent or systematic exposure, consistent with consolidated learning. One-tailed binomial tests were used to assess the proportion of actions exceeding these thresholds, by participant, condition, and age group. To evaluate the differential impact of test type (SP vs DP) on threshold attainment, McNemar tests were conducted to identify significant shifts in recognition proportions between conditions.

Additional descriptive analysis: An additional analysis was performed by calculating, for each player, the total number of actions recognized (out of 40), in both SP and DP conditions. This allowed us to: (1) describe average levels of overall recognition within each group; (2) detect tendencies toward overestimation or underestimation of declarative learning; and (3) explore the extent to which the DP method reduces self-assessment bias.

## 4 Results

### 4.1 . Overall effects of test condition

Mean scores (expressed as percentages) revealed a decline in declarative stability score between the SP and DP conditions across all age groups (Table 3). This decrease was most pronounced among the youngest participants: U-13 (Δ = 12.87), U-15 (Δ = 11.01), and U-18 (Δ = 9.59 percentage points). Shapiro-Wilk tests indicated acceptable approximation to normality at the group level (all *p*>0.050). Visual inspection of Q-Q plots and residual distributions revealed no substantial deviations. Homogeneity of variances was supported (Levene's tests, all *p*> 0.05). Based on these diagnostics, ANOVA was retained as an appropriate analytical approach.

**Table 3:** Distribution of tactical actions by score under SP and DP conditions across age categories (U-13 to U-18).

	**SP**						**DP**					
**Age category**	1	2	3	4	5	Total (%)	1	2	3	4	5	Total (%)
**U-13**	45	5	12	21	17	100	33	3	8	13	8	65
**U-15**	51	14	13	14	8	100	40	5	10	7	4	66
**U-18**	55	26	12	5	2	100	45	11	9	5	1	71

Values correspond to the number of tactical actions rated in each response category (rounded to the nearest unit). Response categories were defined as follows: 1 = I know this action; it was covered during my club's training; 2 = I know this action, but it was not covered during my club's training; 3 = I know this action, but I can't explain it because it was not covered during my club's training; 4 = I have heard about this action, but I can't explain it because it was not covered during training; 5 = I do not know this action; it was not covered during my club's training.

### 4.2 Specific intra- and inter-group comparisons

*Inter-group comparisons (age categories)*: In the SP condition, only the comparison between U-13 and U-18 players was statistically significant (*t* (148) = −2.92, *p* = 0.004, *d* = −0.60, 95% CI [-0.97,−0.19]). In the DP condition, this difference was more pronounced (*t* (148) = −3.83, *p* < 0.001, *d* = −0.78, 95% CI [-1.17,−0.39]). *Intra-group comparisons (SP vs DP):* Significant differences between SP and DP conditions were observed within each age group: U-13: *t*(74) = 7.15, *p* < 0.001, *d* = 1.04, 95% CI [0.67, 1.41]; U-15: *t* (74) = 4.15, *p* < 0.001, *d* = 0.60, 95% CI [0.26, 0.95]; U-18: *t* (74) = 3.58, *p* < 0.001, *d* = 0.52, 95% CI [0.18, 0.86]. Across all groups, SP scores were higher than DP scores.

### 4.3 ANOVA

**Figure 4:**
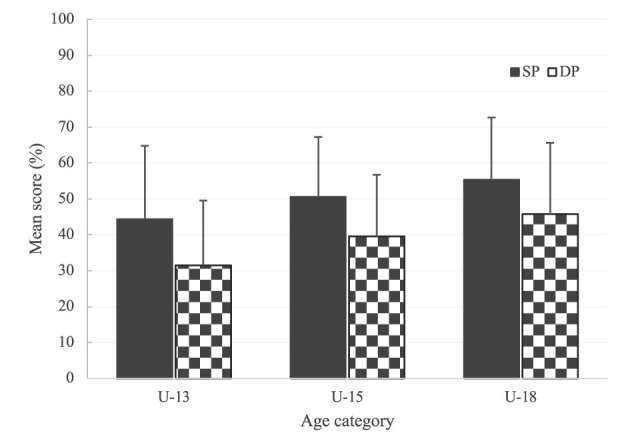
Mean declarative recognition scores (%) in the SP and DP conditions across age categories (U-13, U-15, U-18). Values represent the proportion of tactical actions judged as previously covered during training (out of 40). Error bars indicate standard deviations. DP scores indicate intra-session judgement stability based on consistent responses across two presentations.

The ANOVA revealed a significant main effect of age group, *F* (2, 222) = 9.27, *p* = 0.001, ${\eta^{2}}_{\text{P}}$ = 0.05, 95% CI [0.01, 0.08], and a strong main effect of test condition, *F* (1, 222) = 673.33, *p* < 0.001, ${\eta^{2}}_{\text{P}}$ = 0.75, 95% CI [0.71, 0.78]. A significant interaction between age group and test condition was also found, *F* (2, 222) = 4.87, *p* = 0.008, ${\eta^{2}}_{\text{P}}$ = 0.042, 95% CI [0.003,0.07]. These results indicate statistical differences between age groups, test conditions, and their interaction (Figure 4).

### 4.4 . Dichotomous analysis of declarative integration levels

Moderate threshold (≥ 0.50). The data show an age-related increase in the number of actions recognized as having been addressed in the SP condition. In the DP condition, however, stability was low or even absent among the youngest players. McNemar tests confirmed this trend: U-13 (*p* = 0.001), U-15 (*p* = 0.001), U-18 (*p* = 0.20) (Table 4). High threshold (≥ 0.80). At this more stringent threshold, no action was judged to be fully integrated, regardless of age group or test condition (McNemar: *p* > 0.05 for all groups) (Table 4).

**Table 4:** Proportions of tactical actions perceived as covered during training, according to integration thresholds (≥ 0.50 and ≥ 0.80), by age category.

**Thresholds**	**SP (%)**	**DP (%)**	**McNemar *x*^2^**	**SP (%)**	**DP (%)**	**McNemar *x*^2^**	**SP (%)**	**DP (%)**	**McNemar *x*^2^**
	**U-13**			**U-15**			**U-18**		
≥ 0.50	17	0	0.001^***^	20	3	0.001^***^	27	14	0.27
≥ 0.80	0	0	ns	1	0	ns	1	0	ns

Percentages are rounded to the nearest whole number. Values ≥ 0.50 are rounded up. ns = non-significant. (^*^p < 0.05, ^**^p < 0.01, ^***^p < 0.001).

### 4.5 . Additional descriptive analysis

**Figure 5:**
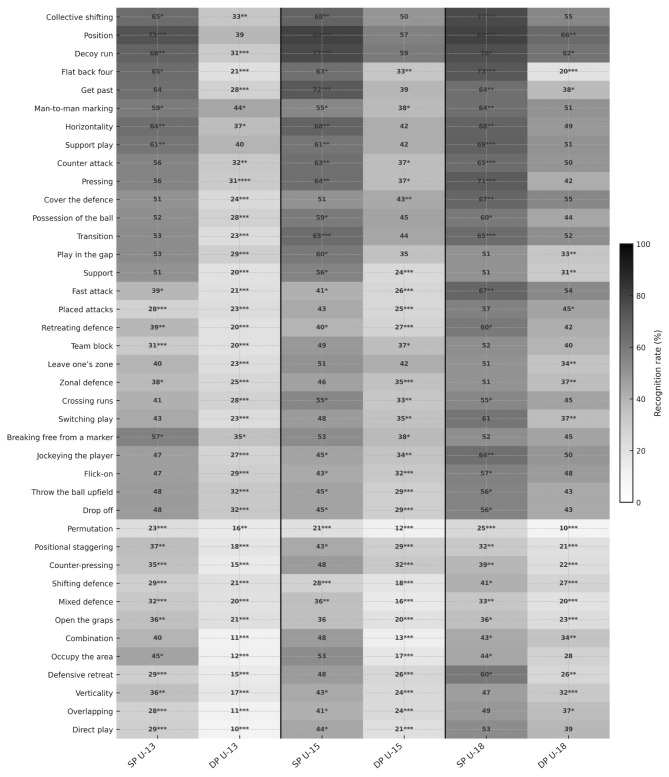
Heatmap of recognition rates for tactical actions across age categories (U-13, U-15, U-18) under SP and DP conditions (*n* = 75 per group). Values represent the percentage of players who indicated that the tactical action had been addressed during training. Darker shading denotes higher recognition. SP = Single-Pass condition; DP = Double-Pass condition. Significance codes: * p < 0.05, ** p < 0.01, *** p < 0.001.

When examined across all 40 actions, stability remained partial overall, with consistently higher scores in the SP condition. U-13 players stabilized an average of 18 actions in SP (45%) and 13 in DP (33%). U-15 players stabilized 20 in SP (51%) and 16 in DP (40%). U-18 players reached 22 in SP (55%) and 18 in DP (45%) (Table 4 and Figure 5). In summary, these results suggest that: (1) immediate recognition (SP) tends to produce an overestimation of learning; (2) judgement stability (DP) reflects only partial consolidation, even among U-18 players; and (3) the overall number of genuinely stabilized tactical actions remains low, raising concerns about the pedagogical effectiveness of current training practices in elite youth development.

## 5 Discussion

The aim of this study was to examine two complementary dimensions of tactical satbility among youth footballers: (1) their ability to consciously recognize tactical situations encountered during training, and (2) short-term stability of their metacognitive judgements.

### 5.1 Toward a more detailed assessment of tactical learning

The present findings support the relevance of a metacognitive approach to evaluating tactical learning in youth footballers. Across all age groups, scores were consistently lower in the DP condition compared to the SP condition, indicating that immediate judgements are less stable when reassessed within the same session. This difference may partly reflect overconfidence in single-pass responses, but should be interpreted cautiously since the design does not directly measure learning magnitude. Importantly, the discrepancy between SP and DP scores was more pronounced in younger players, suggesting that it may reflect age-related differences in verbalisation, attentional control, and judgement calibration rather than genuine disparities in tactical competence. These interpretations are in line with developmental research on metacognitive variability and short-term judgement reliability. The comparatively lower but more consistent scores observed in the DP condition likely may capture a more conservative estimate of declarative accessibility. This finding is consistent with research on judgement stability in short temporal windows (e.g., Serra and Dunlosky, 2010), indicating that repeated assessments can differentiate intuitive recognition from more robust forms of declarative anchoring. Nevertheless, further research is required to determine whether DP performance predicts long-term retention or in-game decision-making, which were not assessed in the present study.

### 5.2 The role of cognitive development

The age-related differences observed in this study suggest a gradual improvement in the ability to recognize and produce more consistent judgements of taught tactical content with increasing cognitive maturity. U-18 players obtained higher scores than U-13 players in both SP and DP conditions, suggesting more advanced declarative accessibility and judgement consistency. These patterns are compatible with developmental models describing the progressive emergence of explicit metacognitive abilities during adolescence (Flavell, 1979; Veenman, 2017). Among U-13 players, greater variability in the DP condition may reflect less stable verbalisation, weaker attentional control, and less consistent judgement calibration at earlier developmental stages. Importantly, these results do not imply lower tactical potential, but rather point to differences in how young players monitor and articulate their knowledge. Taken together, these findings suggest that instructional demands and assessment formats may need to be adapted to learners' cognitive maturity, particularly in the youngest age groups.

### 5.3 Learning partially consolidated

A notable finding is the relatively modest DP performance, even among U-18 players. No age group reached the more stringent threshold of stable recognition (≥ 0.80), and the highest mean values remained below half of the presented actions. Moreover, no signs of attentional disengagement were observed throughout the trials, as indicated by the absence of a significant order effect between passes. This behavioral stability suggests that the double-pass procedure did not induce any notable cognitive fatigue and that participants remained consistently engaged throughout the entire task. While these results do not imply insufficient learning, they suggest that only a limited proportion of taught tactical content is consolidated at a declarative and stable level. Although the intra-session stability observed in this study does not directly demonstrate long-term consolidation, it may be considered a preliminary indicator of cognitive structuring. In other words, increased stability within the same session could reflect an emerging organization of declarative knowledge that may later support durable retention. However, this assumption remains to be empirically tested through longitudinal protocols specifically designed to assess consolidation over time. Several hypotheses may account for this pattern. First, at this developmental stage, a substantial share of tactical knowledge may be integrated implicitly and mobilized in action without explicit verbal recall. Second, current training environments may place greater emphasis on procedural execution rather than reflective verbalisation, potentially constraining the consolidation of explicit declarative representations. Taken together, these observations indicate that pedagogical approaches incorporating more regular metacognitive dialogue, guided reflection, and explicit reference to tactical principles may support deeper declarative anchoring over time. These interpretations should nonetheless be considered with caution, as multiple contextual variables such as coaching methodology, training volume, and seasonal phase were not controlled in the present design and may have influenced the results.

### 5.4 Pedagogical implications

Several pedagogical recommendations emerge from this study: (1) Incorporate regular opportunities for reflective verbalisation such as self-assessment tasks, post-session debriefings, or metacognitive questionnaires to support the stability and explicit evaluation of learning. (2) Align instructional strategies with players' developmental stages: for U-13s, prioritize visual aids and guided tactical games; for U-18s, emphasize tasks involving transfer, self-explanation, and autonomous decision-making. (3) Employ repeated assessment procedures to distinguish between immediate recognition and lasting consolidation, and avoid basing instructional adaptations solely on early or surface-level judgements. (4) Finally, this study appears to support the value of a pedagogy grounded in deep learning and applied metacognition, aimed at fostering tactical knowledge that is explicit, stable, and transferable beyond the immediate context (Hattie and Donoghue, 2016).

### 5.5 Limitations and perspectives

This study presents several limitations. First, recognition was assessed solely through a declarative recognition task. Complementary methods such as open-ended verbalisation, qualitative interviews, or performance-based assessments could enrich the understanding of how tactical knowledge is expressed. Although the recognition-based format provides a controlled way to probe declarative anchoring, the absence of external validation and diagnostic accuracy measures (e.g., hits, false alarms, d′) represents a methodological limitation of the present design. From a validity standpoint, however, the task employed in this study closely aligns with the cognitive demands of real-game situations, thereby supporting its content validity. Furthermore, the correspondence between metacognitive judgements and actual performance outcomes suggests satisfactory criterion validity. These findings are consistent with studies grounded in signal detection theory applied to sport performance (e.g., Oudejans and Pijpers, 2009; Tenenbaum et al., 2013) which emphasize the relevance of approaches sensitive to attentional and decision-making fluctuations in dynamic environments. Future studies could include coach-verified exposure logs and optional tactical justifications to enhance construct validity. Second, although the experimental protocol was rigorous, it was conducted outside the natural training environment. Further ecological validation in real-game contexts is required to confirm the applicability of the findings. Contextual and training-related variables (e.g., coaching approach, seasonal period) were not controlled in the current design. These factors may substantially influence recognition stability and should be treated as covariates or control variables in future research. Third, the study also did not control for instructional quality, including coaching style and the frequency of tactical reinforcement. Future research should explore the relationship between the stability of metacognitive judgements and actual in-game performance. It would also be valuable to examine the longitudinal effects of metacognitive feedback mechanisms implemented during training. Despite these limitations, this work makes an original contribution by articulating the metacognitive dimensions of tactical stability within a controlled experimental framework. Theoretically, it advances our understanding of the stability of declarative accessibility from a metacognitive perspective. Methodologically, it proposes a reproducible protocol for identifying explicit stability indicators. Practically, it provides concrete avenues for designing training programmes that integrate self-regulation and metacognitive feedback into the development of young players.

## 6 Conclusion

The present findings show that explicit recognition of taught tactical content remains partial and often superficial, particularly among younger players. The generally low stability of metacognitive judgements indicates that immediate recognition does not necessarily reflect cognitive anchoring. Importantly, these results concern the short-term explicit accessibility of tactical concepts and do not allow conclusions about their long-term consolidation or retention. These results suggest that exposure to tactical content alone is insufficient to ensure stable access in memory. From a practical perspective, coaches may benefit from integrating brief moments of reflective questioning and short re-exposures within the same training session, to verify whether players can reliably recognize previously addressed content beyond immediate intuition. Such adjustments may help align instructional pacing with players' developmental profiles. Finally, developmental variability in stability highlights the need for assessment procedures that account for differences in cognitive maturation. In this regard, the double-pass method could serve as a valuable tool for the longitudinal monitoring of tactical and metacognitive development in young players. When implemented repeatedly within training programmes, it may offer coaches a personalized means to track tactical reasoning and information-processing quality over time. Future research should examine how these patterns evolve over time and integrate complementary qualitative indicators of tactical knowledge internalization.

## Data Availability

The data analyzed in this study is subject to the following licenses/restrictions: The data used in this study are available for verification by the journal's editorial board or peer reviewers upon request. However, due to the sensitive nature of the information collected and the fact that participants did not provide consent for public sharing, the data cannot be made publicly available. Any request for access will be considered in accordance with ethical requirements and the need to protect participant confidentiality. Requests to access these datasets should be directed to zoudji.bachir@uphf.fr.

## References

[B1] Abernethy B. Baker J. Côté J. (2005). Transfer of pattern recall skills may contribute to the development of sport expertise. Appl. Cogn. Psychol. 19, 705–718. doi: 10.1002/acp.1102

[B2] Acoumambo D. Zoudji B. (2023). Development of Tactical Lexicon Among Young Footballers 12 to 15 Years. HAL. Retrieved from: https://uphf.hal.science/hal-04493883v1 (Accessed April 17, 2025).

[B3] Ade J. Fitzpatrick J. Bradley P S. (2016). High-intensity efforts in elite soccer matches and associated movement patterns, technical skills and tactical actions. Information for position-specific training drills. J. Sports Sci. 34, 2205–2214. doi: 10.1080/02640414.2016.1217343 27537056

[B4] Anderson J. R. (1982). Acquisition of cognitive skill. Psychol. Rev. 89, 369–406. doi: 10.1037/0033-295X.89.4.369

[B5] Aranda-Malavés R. Casals C. A. Gonzalez Rodenas J. Tudela-Desantes A. De Matías-Cid P. Moltó-Llorens M. . (2024). Tactical analysis of direct attack from the English Premier League and Spanish La Liga. Front. Sports Act. Living 6:1473311. doi: 10.3389/fspor.2024.1473311 39435364 PMC11491368

[B6] Araújo D. Davids K. Hristovski R. (2006). The ecological dynamics of decision making in sport. Psychol. Sport Exerc. 7, 653–676. doi: 10.1016/j.psychsport.2006.07.002

[B7] Beernaerts J. De Baets B. Lenoir M. Van De Weghe N. (2020). Spatial movement pattern recognition in soccer based on relative player movements. PLoS ONE 15:e0227746. doi: 10.1371/journal.pone.0227746 31945108 PMC6964894

[B8] Beilock S. L. Carr T. H. (2001). On the fragility of skilled performance: what governs choking under pressure? J. Exp. Psychol. Gen. 130, 701–725. doi: 10.1037/0096-3445.130.4.701 11757876

[B9] Chawla S. Estephan J. Gudmundsson J. Horton M. (2017). Classification of passes in football matches using spatiotemporal data. ACM Trans. Spat. Algorithms Syst. 3, 1–30. doi: 10.1145/3105576

[B10] Cho H. Ryu H. Song M. (2022). Pass2vec: analyzing soccer players' passing style using deep learning. Int. J. Sports Sci. Coach. 17, 355–365. doi: 10.1177/17479541211033078

[B11] Chow J. Y. Kee Y. H. (2022). An ecological dynamics perspective to learning. Asian J. Sport Exerc. Psychol. 2, 1–2. doi: 10.1016/j.ajsep.2022.04.004

[B12] Costa I. T. D. Garganta J. Greco P. J. Mesquita I. (2011). Proposta de avaliação do comportamento tático de jogadores de futebol baseada em princípios fundamentais do jogo. Motriz Rev. Educ. Fís. 17, 511–524. doi: 10.1590/S1980-65742011000300014

[B13] Deprez D. Buchheit M. Fransen J. Pion J. Lenoir M. Philippaerts R. M. . (2015). A longitudinal study investigating the stability of anthropometry and soccer-specific endurance in pubertal high-level youth soccer players. J. Sports Sci. Med. 14, 418–426. 25983593 PMC4424473

[B14] Díaz Losquiño T. Solà Santesmases J. (2018). Análisis funcional del desmarque en el fútbol [Functional analysis of losing your marker in football]. Apunts Educ. Física Deport. 132, 60–71. doi: 10.5672/apunts.2014-0983.es.(2018/2).132.05

[B15] Dunlosky J. Metcalfe J. (2009). Metacognition (p. ix, 334). Thousand Oaks, CA: Sage Publications, Inc.

[B16] Fernandes T. Camerino O. Castañer M. (2021). T-pattern detection and analysis of football players' tactical and technical defensive behaviour interactions: insights for training and coaching team coordination. Front. Psychol. 12:798201. doi: 10.3389/fpsyg.2021.798201 34938248 PMC8685770

[B17] Fernandez-Navarro J. Fradua L. Zubillaga A. Ford P. R. McRobert A. P. (2016). Attacking and defensive styles of play in soccer: analysis of Spanish and English elite teams. J. Sports Sci. 34, 2195–2204. doi: 10.1080/02640414.2016.1169309 27052355

[B18] Flavell J. H. (1979). Metacognition and cognitive monitoring: a new area of cognitive–developmental inquiry. Am. Psychol. 34, 906–911. doi: 10.1037/0003-066X.34.10.906

[B19] Fleming S. M. Dolan R. J. (2012). The neural basis of metacognitive ability. Philos. Trans. R. Soc. Lond. B. Biol. Sci. 367, 1338–1349. doi: 10.1098/rstb.2011.0417 22492751 PMC3318765

[B20] Forcher L. Forcher L. Wäsche H. Jekauc D. Woll A. Gross T. . (2023). Is ball-possession style more physically demanding than counter-attacking? The influence of playing style on match performance in professional soccer. Front. Psychol. 14:1197039. doi: 10.3389/fpsyg.2023.1197039 37484109 PMC10361297

[B21] Frank D. J. Kuhlmann B. G. (2017). More than just beliefs: experience and beliefs jointly contribute to volume effects on metacognitive judgments. J. Exp. Psychol. Learn. Memory Cogn. 43, 680–693. doi: 10.1037/xlm0000332 27709983

[B22] González-Víllora S. Serra-Olivares J. Pastor-Vicedo J. C. Da Costa I. T. (2015). Review of the tactical evaluation tools for youth players, assessing the tactics in team sports: football. SpringerPlus 4:663. doi: 10.1186/s40064-015-1462-0 26558166 PMC4630321

[B23] Gregory S. Robertson S. Aughey R. Duthie G. (2022). The influence of tactical and match context on player movement in football. J. Sports Sci. 40, 1063–1077. doi: 10.1080/02640414.2022.2046938 35254225

[B24] Gumusdag H. Egesoy H. Sahbudak E. (2025). Decision making in sport: the role of attention, prioritisation and memory. Psychol. Psychol. Res. Int. J. 10, 1–6. doi: 10.23880/pprij-16000454

[B25] Håland E. M. Wiig A. S. Stålhane M. Hvattum L. M. (2020). Evaluating passing ability in association football. IMA J. Manag. Math. 31, 91–116. doi: 10.1093/imaman/dpz004

[B26] Han B. Li X. Li Y. Liu T. (2024). Analysis of attacking corner kick strategies in the Chinese Women's Super League 2019. Proc. Inst. Mech. Eng. Part P J. Sports Eng. Technol. 17543371241265595. doi: 10.1177/17543371241265595

[B27] Harvey S. Jarrett K. (2014). A review of the game-centred approaches to teaching and coaching literature since 2006. Phy. Educ. Sport Pedagog. 19, 278–300. doi: 10.1080/17408989.2012.754005

[B28] Hattie J. A. C. Donoghue G. M. (2016). Learning strategies: a synthesis and conceptual model. Npj Sci. Learn. 1:16013. doi: 10.1038/npjscilearn.2016.13 30792898 PMC6380372

[B29] Höner O. Feichtinger P. (2016). Psychological talent predictors in early adolescence and their empirical relationship with current and future performance in soccer. Psychol. Sport Exerc. 25, 17–26. doi: 10.1016/j.psychsport.2016.03.004

[B30] Hsu C.-C. Sandford B. A. (2007). The Delphi Technique: Making Sense of Consensus. Massachusetts, MA: University of Massachusetts

[B31] Izquierdo J. M. Marqués-Jiménez D. Redondo J. C. (2023). Running demands and tactical individual actions of wingers appear to depend on the playing formations within an amateur football team. Sci. Rep. 13:8927. doi: 10.1038/s41598-023-36157-6 37264075 PMC10235107

[B32] Kannekens R. Elferink-Gemser M. T. Visscher C. (2009). Tactical skills of world-class youth soccer teams. J. Sports Sci. 27, 807–812. doi: 10.1080/02640410902894339 19437183

[B33] Konefał M. Chmura P. Zaja̧c T. Chmura J. Kowalczuk E. Andrzejewski M. (2019). A new approach to the analysis of pitch-positions in professional soccer. J. Hum. Kinet. 66, 143–153. doi: 10.2478/hukin-2018-0067 30988848 PMC6458575

[B34] Koriat A. (1997). Monitoring one's own knowledge during study: A cue-utilization approach to judgments of learning. J. Exp. Psychol. Gen. 126, 349–370. doi: 10.1037//0096-3445.126.4.349

[B35] Leontijević B. Tomić L. Šmrkić M. Nikolić D. Janković A. (2021). Analysis of attack tactics of football teams in the champions league for the period from 2014 to 2019. Fiz Kult. 75, 153–160. doi: 10.5937/fizkul75-30145

[B36] Light R. (2004). Coaches' experiences of game sense: opportunities and challenges. Phy. Educ. Sport Pedagog. 9, 115–131. doi: 10.1080/1740898042000294949

[B37] Mackenzie R. Cushion C. (2013). Performance analysis in football: a critical review and implications for future research. J. Sports Sci. 31, 639–676. doi: 10.1080/02640414.2012.746720 23249092

[B38] Matsubara H. Kida N. Nomura T. (2022). A study on support play in soccer games: relationship between player positioning and zone. Adv. Phys. Educ. 12, 271–282. doi: 10.4236/ape.2022.123021

[B39] McPherson S. L. (1994). The development of sport expertise: mapping the tactical domain. Quest 46, 223–240. doi: 10.1080/00336297.1994.10484123

[B40] Memmert D. Raabe D. (2018). Data Analytics in Football: Positional Data Collection, Modelling and Analysis, 1^*st*^ *Edn*. London: Routledge doi: 10.4324/9781351210164

[B41] Miyamoto M. Kaneki Y. (2019). “Measuring tactics of taking the ball away from defenders in the Japanese football league,” in Advances in Social and Occupational Ergonomics, Vol. 792, ed. R. H. M. Goossens (Springer International Publishing), 336–348. doi: 10.1007/978-3-319-94000-7_35

[B42] Muñoz O. Monroy R. Cañete-Sifuentes L. Ramirez-Marquez J. E. (2024). Automated discovery of successful strategies in association football. Appl. Sci. 14:1403. doi: 10.3390/app14041403

[B43] Moher D. Liberati A. Tetzlaff J. Altman D. G. The PRISMA Group. (2009). Preferred reporting items for systematic reviews and meta-analyses: the PRISMA statement. PLoS Med. 6:e1000097. doi: 10.1371/journal.pmed.1000097 21603045 PMC3090117

[B44] Moreira P. E. D. Sousa R. B. E. Morales J. C. P. Greco P. J. Arroyo M. P. M. Praça G. M. (2020). Comportamiento táctico de jugadores de fútbol de diferentes posiciones, durante una temporada deportiva (Tactical behaviour of soccer players from different playing positions throughout a season). Retos 39, 1–6. doi: 10.47197/retos.v0i39.75970

[B45] Nunes N. A. Gonçalves B. Coutinho D. Nakamura F. Y. Travassos B. (2022). How playing area dimension and number of players constrain football performance during unbalanced ball possession games. Int. J. Sports Sci. Coach. 16, 334–343. doi: 10.1177/1747954120966416

[B46] O'Connor D. Larkin P. Williams A. M. (2018). Observations of youth football training: how do coaches structure training sessions for player development? J. Sports Sci. 36, 39–47. doi: 10.1080/02640414.2016.1277034 28065123

[B47] Oudejans R. R. D. Pijpers J. R. (2009). Training with anxiety has a positive effect on expert perceptual–motor performance under pressure. Quar. J. Exp. Psychol. 62, 1631–1647. doi: 10.1080/17470210802557702 19123115

[B48] Palucci Vieira L. H. Carling C. Barbieri F. A. Aquino R. Santiago P. R. P. (2019). Match running performance in young soccer players: a systematic review. Sports Med. 49, 289–318. doi: 10.1007/s40279-018-01048-8 30671900

[B49] Plakias S. Tsatalas T. Armatas V. Tsaopoulos D. Giakas G. (2024). Tactical situations and playing styles as key performance indicators in soccer. J. Funct. Morphol. Kinesiol. 9:88. doi: 10.3390/jfmk9020088 38804454 PMC11130910

[B50] Quartiroli A. (2025). The Delphi technique in sport, exercise, and performance psychology: extensive scoping review, insights, and recommendations for scholars. Sport, Exerc. Perform. Psychol. 14, 57–77. doi: 10.1037/spy0000364

[B51] Reilly T. Williams A. M. Nevill A. Franks A. (2000). A multidisciplinary approach to talent identification in soccer. J. Sports Sci. 18, 695–702. doi: 10.1080/02640410050120078 11043895

[B52] Sánchez-López R. Echeazarra I. Castellano J. (2022). Systematic review of declarative tactical knowledge evaluation tools based on game-play scenarios in soccer. Qual. Quant. 56, 2157–2176. doi: 10.1007/s11135-021-01204-9

[B53] Schmidt R. A. Lee T. D. (2011). Motor Control and Learning: A Behavioral Emphasis, 5th ed (p. ix, 581). Champaign, IL: Human Kinetics.

[B54] Schmidt R. A. Wrisberg C. A. (2008). Motor Learning and Performance: A Situation-Based Learning Approach, 4th Edn. (p. xx, 395). Champaign, IL: Human Kinetics.

[B55] Serra M. J. Dunlosky J. (2010). Metacomprehension judgements reflect the belief that diagrams improve learning from text. Memory 18, 698–711. doi: 10.1080/09658211.2010.506441 20730677

[B56] Shea N. Boldt A. Bang D. Yeung N. Heyes C. Frith C. D. (2014). Supra-personal cognitive control and metacognition. Trends Cogn. Sci. 18, 186–193. doi: 10.1016/j.tics.2014.01.006 24582436 PMC3989995

[B57] Sweller J. (2011). “Cognitive Load Theory,” in Psychology of Learning and Motivation, eds. J. P. Mestre and B. H. Ross (Amsterdam: Elsevier), 37–76. doi: 10.1016/B978-0-12-387691-1.00002-8

[B58] Teixeira J. E. Forte P. Ferraz R. Branquinho L. Silva A. J. Monteiro A. M. . (2022). Integrating physical and tactical factors in football using positional data: a systematic review. PeerJ 10:e14381. doi: 10.7717/peerj.14381 36405022 PMC9671036

[B59] Tenenbaum G. Basevitch I. Gershgoren L. Filho E. (2013). Emotions–decision-making in sport: theoretical conceptualization and experimental evidence. Int. J. Sport Exerc. Psychol. 11, 151–168. doi: 10.1080/1612197X.2013.773687

[B60] Van Merriënboer J. J. G. Sweller J. (2010). Cognitive load theory in health professional education: design principles and strategies: cognitive load theory. Med. Educ. 44, 85–93. doi: 10.1111/j.1365-2923.2009.03498.x 20078759

[B61] Veenman M. V. J. (2017). Assessing metacognitive deficiencies and effectively instructing metacognitive skills. Teach. Coll. Rec. Voice Schol. Educ. 119, 1–20. doi: 10.1177/016146811711901303

[B62] Veenman M. V. J. Van Hout-Wolters B. H. A. M. Afflerbach P. (2006). Metacognition and learning: conceptual and methodological considerations. Metacogn. Learn. 1, 3–14. doi: 10.1007/s11409-006-6893-0

[B63] Wang S. H. Qin Y. Jia Y. Igor K. E. (2022). A systematic review about the performance indicators related to ball possession. PLoS ONE 17:e0265540. doi: 10.1371/journal.pone.0265540 35298562 PMC8929629

[B64] Williams A. M. Hodges N. J. (2005). Practice, instruction and skill acquisition in soccer: challenging tradition. J. Sports Sci. 23, 637–650. doi: 10.1080/02640410400021328 16195012

[B65] Zimmerman B. J. (2002). Becoming a self-regulated learner: an overview. Theory Pract. 41, 64–70. doi: 10.1207/s15430421tip4102_2

[B66] Zoudji B. Thon B. (2003). Expertise and implicit memory: differential repetition priming effects on decision making in experienced and inexperienced soccer players. Int. J. Sport Psychol. 34, 189–207.

[B67] Zoudji B. Thon B. Debû B. (2010). Efficiency of the mnemonic system of expert soccer players under overload of the working memory in a simulated decision-making task. Psychol. Sport Exerc. 11, 18–26. doi: 10.1016/j.psychsport.2009.05.006

